# Clinical differences between toric intraocular lens (IOL) and monofocal intraocular lens (IOL) implantation when myopia is determined as target refraction

**DOI:** 10.1186/s12886-021-01966-8

**Published:** 2021-05-08

**Authors:** Da Young Shin, Ho Sik Hwang, Hyun Seung Kim, Man Soo Kim, Eun Chul Kim

**Affiliations:** 1 Department of Ophthalmology, Eunpyeong St. Mary’s Hospital, College of Medicine, The Catholic University of Korea, Seoul, Republic of Korea; 2 Department of Ophthalmology, Yeouido St. Mary’s Hospital, College of Medicine, The Catholic University of Korea, Seoul, South Korea; 3 Department of Ophthalmology, Seoul St. Mary’s Hospital, College of Medicine, The Catholic University of Korea, Seoul, South Korea; 4 Department of Ophthalmology, Bucheon St. Mary’s Hospital, Catholic University of Korea, #327 Sosa-ro, 14647 Bucheon, Korea

**Keywords:** Toric intraocular lens implantation, Corneal astigmatism, Tecnis ZCT toric IOL, Myopia, Near visual acuity, Distant visual acuity

## Abstract

**Background:**

The aim of this study is to analyze and compare the clinical results of toric intraocular lens (IOL) and monofocal IOL implantation when the target refraction value is -3 diopter (D) in cataract patients with corneal astigmatism > 1.5 diopters (D).

**Methods:**

We performed a retrospective chart review for patients with corneal astigmatism > 1.5D who underwent cataract surgery and their target refraction is -3D. 100 eyes (100 patients; monofocal IOL, 60; toric IOL, 40) were enrolled in the current study. Near and distant uncorrected visual acuity (UCVA), corrected VA, spherical equivalent and refractive, corneal astigmatism were evaluated before and after surgery.

**Results:**

The near UCVA of the toric IOL group (0.26 ± 0.33) after cataract surgery was significantly better than that of the monofocal IOL group (0.48 ± 0.32) (*p* = 0.030). The distant UCVA of the toric IOL group (0.38 ± 0.14) was also significantly better than that of the monofocal IOL group (0.55 ± 0.22) (*p* = 0.026). Best-corrected visual acuity (*p* = 0.710) and mean spherical equivalent (*p* = 0.465) did not show significant differences between the toric IOL group and the monofocal IOL group. In the toric IOL group, postoperative refractive astigmatism was − 0.80 ± 0.46D and postoperative corneal astigmatism was − 1.50 ± 0.62D, whereas the corresponding values in the monofocal IOL group were − 1.65 ± 0.77D and − 1.45 ± 0.64D; residual refractive astigmatism was significantly lower with toric IOL implantation compared with monofocal IOL implantation (*p* = 0.001). There were no postoperative complications.

**Conclusions:**

When myopic refraction such as -3D was determined as the target power in patients with corneal astigmatism, toric IOL implantation led to excellent improvement in both near and distant UCVA.

## Background

Multiple advances in cataract surgical techniques are being employed to improve the quality of postoperative visual acuity (VA). Corneal astigmatism reduces uncorrected visual acuity (UCVA) after surgery, which is one of the main reasons for increased dependence on corrective lenses [1]. Studies conducted in China, Southwest Asia and Europe have reported that 22.1–26.2 % of the population have corneal astigmatism > 1.5 diopters (D) [2–4]. Accordingly, various methods, such as arcuate incision using limbal relaxing incision (LRI) and femtosecond laser, toric intraocular lens (IOL) implantation, and photorefractive keratectomy, have been studied for correction of corneal astigmatism in cataract surgery [5]. However, it is difficult to simultaneously perform photorefractive keratectomy with cataract surgery, and expensive machines are needed for arcuate incision using femtosecond laser; therefore, LRI and toric IOL implantation are usually preferred. Toric IOLs implantation has been reported to be more effective than LRI [6]. Toric lOLs has become the most recommended method for correcting astigmatism in cataract patients.

The advantages of toric lenses over conventional IOLs have already been studied. Pineda et al. [7] reported that the toric IOLs provide an additional 10 to 20 quality adjusted life years compared with conventional IOLs with and without intraoperative refractive correction. However, most of these studies have done to patient who underwent surgery for emmetropic target refraction. In fact, ophthalomologists tend not to recommend toric IOLs for patients who want − 3D as their target refraction power. This is because patients who have − 3D as refractive power should always wear spectacle for distance vision such as driving, walking, watching television, so the remaining astigmatism can be corrected together with this spectacle. However, these patients who have − 3D as their refractive power do not always wear spectacles at home and they often take them off when taking a shower or reading a book. The uncorrected visual acuity can be quite important considering it.

In the present study, we compared the postop uncorrected visual acuity of near and distant between the toric IOL group and the monofocal IOL group, when myopic refraction such as -3D was determined as the target power in cataract patients with corneal astigmatism.

## Methods

### Patients

This study retrospectively reviewed 100 eyes (60 with monofocal IOL; 40 with toric IOL) of 100 patients who underwent cataract surgery with preoperative corneal astigmatism of > 1.5D in Bucheon St. Mary’s Hospital, Bucheon, South Korea from January 2014 to December 2015. It was approved by Institutional review and Ethics Board of Seoul St. Mary’s Hospital and we followed all relevant tenets of the Declaration of Helsinki.

The main inclusion criteria were patients who have age- related cataract with corneal astigmatism over 1.5 Diopter.

The study’s exclusion criteria included a history of any ocular disease affecting visual acuity such as infection, trauma, glaucoma, intraocular surgery; a history of retinal disease such as retinal detachment, macular hole, epiretinal membrane, vitreous hemorrhage; an eye with irregular astigmatism such as keratoconus and keratoectasia.

### Preoperative examination

The following were evaluated as part of the preoperative examination: detailed medical history; slit lamp microscopy and fundus examination, distant UCVA and best-corrected visual acuity (BCVA); autorefraction); and corneal topography (Pentacam, Oculus GmBH, Wetzlar, Germany). The axial length used was the mean value determined from two measurements with the IOL Master (Carl Zeiss Meditec Inc., Dublin, CA, USA) twice, and IOL power was calculated using the SRK/T formula. Patients with corneal astigmatism of > 1.5 D on both in autorefraction and corneal topography were included as subjects. Implantation of a toric versus monofocal IOL was decided according to the patients’s wishes, after the cost of a toric IOL was explained to them. Patients with irregular astigmatism, pupil size < 5.5 mm, retinal abnormalities, and other ophthalmic or systemic diseases that might have affected the corneal topographic mapping or VA were excluded.

### Toric and monofocal IOLs

The Tecnis® ZCB IOL (Abbott Medical Optics, Santa Ana, CA, USA) was used as a monofocal IOL, and the Tecnis® ZCT toric IOL (Abbott Medical Optics) as a astigmatic IOL. To determine the IOL power, two values were measured, and averaged, with an Aviso® (Quantel Medical, Clemont-Ferrand, France) and using the ultrasonic bonding method and IOL Master® (Carl Zeiss Meditiec, Jena, Germany) based on the principle of partial coherence interferometry. Measurements were performed by a skilled examiner. IOL power was calculated by setting the target refraction to -3 D using the SRK/T formula. The estimated A-Constants were 118.8 for A-scan and 119.3 for IOL Master. IOL cylinder power and alignment axis were measured using a web-based calculation program (http://www.amoeasy.com/calc), which considers keratometry as well as surgically induced astigmatism, which was set to 0.5.

### Surgical methods

The patient, once seated, was asked to look straight into the slit lamp microscope, which allowed the calculation of the long-distance fixation point. Reference markers were placed at the 3 and 9 o’clock directions on the corneal limbus using a toric reference corneal marker (AE-2793 S, ASICO) just before the surgery. The intended axis of IOL placement was marked on the cornea with a toric axis marker (K3-7910, Katena) with the patient lying down during surgery. All procedures were performed by the same surgeon (Eun Chul Kim), and a 2.75-mm clear corneal incision was made in the direction of the steep axis of astigmatism. Sodium hyaluronate 1.0 % (Hyal Plus, LG Life Science, Seoul, Korea) was used for stabilizing anterior chamber. A continuous curvilinear capsulotomy was made using Inamura capsulorhexis forceps (Duckworth & Kent Ltd., Baldock, UK). Hydrodissection and hydrodelineation were performed by a balanced salt solution. Endocapsular phacoemulsification was performed using 2.75-mm-sized phaco-tips and infusion/aspiration cannulas the Infinity Vision System® (Alcon Laboratories Inc., Fort Worth, TX, USA) and OZil™ Torsional Handpiece (Alcon Laboratories Inc.). A clear preloaded IOL (Tecnis® ZCB00 IOL or Tecnis® ZCT toric IOL) was implanted in the capsular bag. The remaining viscoelastic materials behind the IOL were removed after implantation of IOL. In the case of toric IOLs, the IOL axis was rotated until it was positioned parallel to the previously marked. The operation was terminated without corneal suture.

### Postoperative assessment

Distance (6.0 m) UCVA and BCVA, auto-refraction, keratometry and slit lamp examinations were evaluated postoperatively on day 1, week 1, and months 1 and 2. Distance VA was assessed at 6.0 m using a Snellen chart. Near visual acuity was assessed using the Rosenbaum near vision card at 33-cm near-distance after 2 months of surgery. All VA values are expressed as logarithm of the minimum angle of resolution (LogMAR). In the group that underwent toric IOL implantation, pupil of patients were dilated to visualize the axis of rotation 2 months post-surgery.

### Statistical analysis

Descriptive statistics were calculated as means and standard deviation and a chi-squared test was used to compare categorical variables. The Kolmogorov–Smirnov test was used to test for normality, and the independent t-test was used for intergroup comparisons. A value of *P* < 0.05 indicated statistical significance. All statistical analyses were performed using SPSS for Windows (v. 24.0, IBM Corporation, Armonk, NY, USA).

## Results

There were no statistically significant intergroup (toric vs. monofocal) differences with respect to age, sex, preoperative refractive astigmatism, or corneal astigmatism (Table 1).

**Table 1:** Preoperative demographic and biometric data of the subjects (*n*=60)

Parameter	Group		*p* value
	Monofocal IOL	Toric IOL	
Eyes (n)	60	40	
IOL model	Technis ZCB00	Technis ZCT toric	
Sex (M/F)	23/37	12/28	
Age (years)	64.4±4.64	62.7±11.3	0.542
Mean UCVA (logMAR)	0.65±0.29	0.72±0.28	0.445
Mean BCVA (logMAR)	0.40±0.35	0.36±0.27	0.947
Mean refractive cylinder(D)	1.84±0.35	2.18±1.00	0.277
Mean keratometric cylinder(D)	1.82±0.35	1.76±1.07	0.102

Values are presented as mean±standard deviation unless otherwise indicated

*IOL* intraocular lens, *D* diopters

**Fig. 1:**
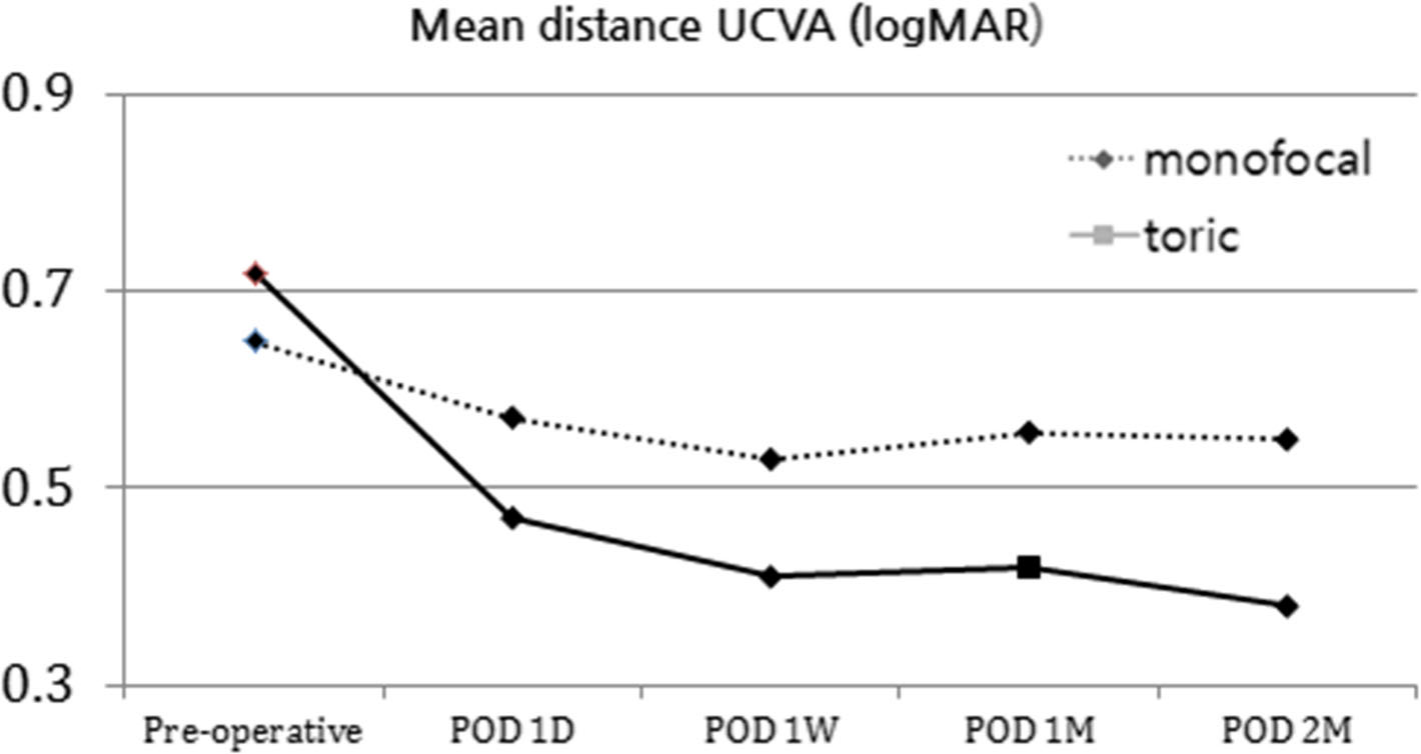
Pre and postoperative distant UCVA (logMAR) of treatment groups (monofocal, toric). UCVA, uncorrected distance visual acuity; POD, postoperative day

**Fig. 2:**
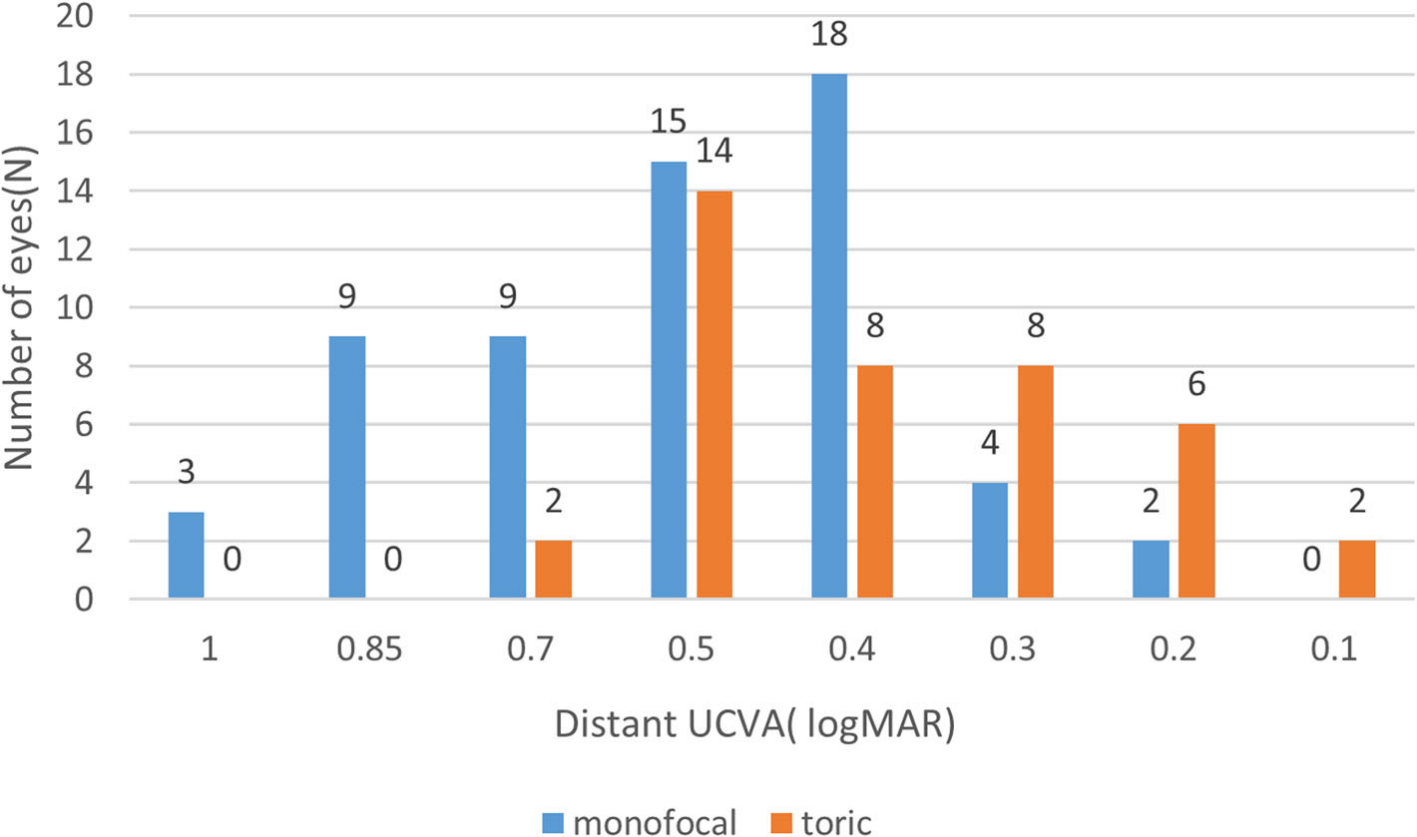
Number of eyes according to postoperative distant UCVA (logMAR) in two treatment groups(monofocal, toric IOL). UCVA, uncorrected distance visual acuity; POD, postoperative day

**Fig. 3:**
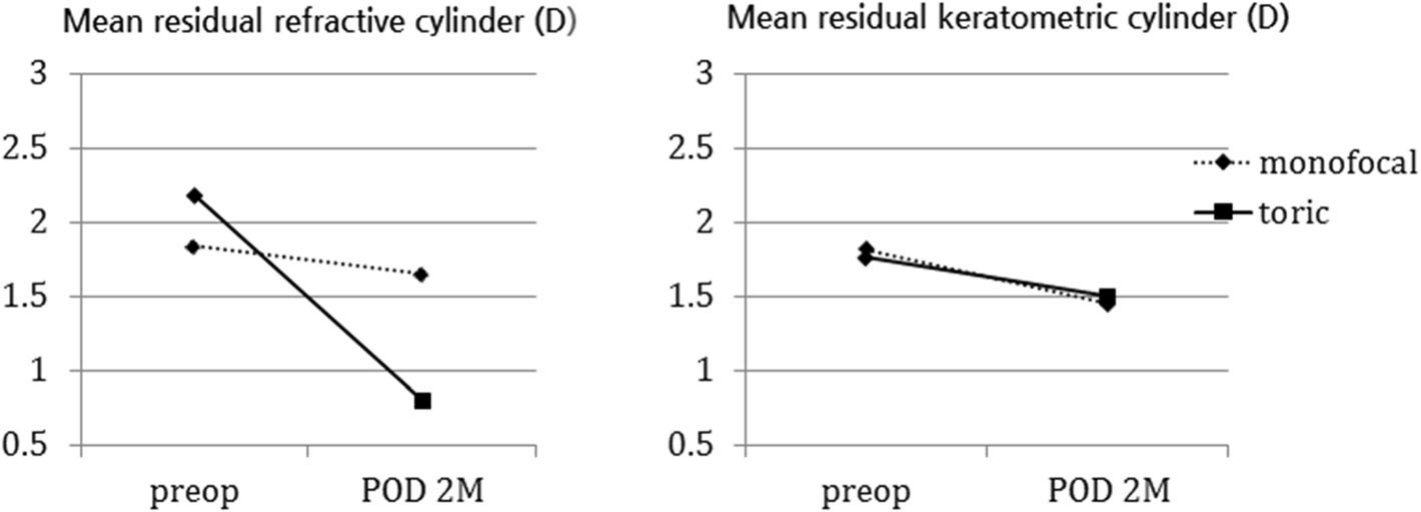
Pre and postoperative mean residual refractive cylinder(D) and mean residual keratometric cylinder(D) of treatment groups (monofocal, toric). POD, postoperative day

There were no significant differences between two groups in spherical equivalent (*p* = 0.46, Table 3). UCVA at a distance improved in both groups after cataract surgery, reaching 0.55 ± 0.22 in the monofocal group and 0.38 ± 0.14 in the toric (Fig. 1). The UCVA (0.26 ± 0.33) at a distance of 33 cm in the toric IOL group was significantly better than the UCVA (0.48 ± 0.32) in the monofocal IOL group 2 months after cataract surgery (*p* = 0.030). The distance UCVA in the toric IOL group (0.38 ± 0.14) was significantly better than the monofocal IOL group (0.55 ± 0.22) (*p* = 0.026, Fig. 2; Table 2). There were no significant differences between groups in distance BCVA (*p* = 0.710, Fig. 3; Table 2).

**Table 2:** Postoperative visual acuity(logMAR) according to treatment group(monofocal, toric)

Parameter	Group		*p* value
	Monofocal IOL	Toric IOL	
Mean distance UCVA	0.55±0.22	0.38±0.14	0.026
Mean distance BCVA (logMAR)	0.11±0.14	0.08±0.08	0.710
Mean near UCVA (logMAR)	0.48±0.32	0.26±0.33	0.030

Values are presented as mean±standard deviation unless indicated otherwise

*IOL* intraocular lens, *UCVA* uncorrected visual acuity, *BCVA* best-corrected visual acuity

Spherical equivalent, cornea astigmatism, and refractive astigmatism were analyzed with autorefraction 2 months after surgery. Mean spherical equivalent values are − 3.05 ± 0. 63 D in the monofocal and − 3.23 ± 0. 85 D in the toric. Corneal astigmatism values in the monofocal and toric IOL groups were 1.45 ± 0. 64 D and 1.50 ± 0.62 D, respectively, at that time. While there there were no statistically significant differences in spherical equivalent (*p* = 0.465) and corneal astigmatism (*p* = 0.626), the refractive astigmatism in the toric IOL groups (0.80 ± 0.46 D) was significantly lower than in the monofocal groups (1.65 ± 0.77D, *p* = 0.001; Fig. 2; Table 3).

**Table 3:** Preoperative and postoperative mean residual refractive cylinder (D) and mean residual keratometric cylinder (D) according to treatment group(monofocal, toric)

Parameter	Group		*p* value
	Monofocal IOL	Toric IOL	
Mean residual refractive cylinder (D)	1.65±0.77	0.80±0.46	0.001
Mean residual keratometric cylinder (D)	1.45±0.64	1.50±0.62	0.626

Values are presented as mean±standard deviation unless otherwise indicated

*IOL* intraocular lens, *D* diopters

There were no complications, such as rupture of the capsule, occurred during the surgeries and no postoperative complications were reported.

## Discussion

A number of studies have reported the influence of astigmatism on visual function. Several studies have shown that retaining some of myopic astigmatism of against-the-rule would increases the depth of focus and increases near VA, without decreasing distance VA, in case the astigmatism cannot be corrected perfectly when cataract surgery for emmetropia is conducted [1, 8–12]. However, studies are increasing about adverse effects of astigmatism on visual function. Kamiya et al. [13] reported that uncorrected distant VA was better in eyes with less astigmatism. Wolffsohn et al. [14] reported that even if the uncorrected astigmatism is as small as 1D, it would significantly diminish the VA and affect the patient’s independence and quality of life. Wiggins and Daum [15] reported that patients with 0.50D of uncorrected cylinder complained of more eye fatigue and discomfort than well corrected patients when using a visual display terminal. Rosenfield et al. [16] suggested that small astigmatism should be corrected for comfort when working on a computer. Kazuhiro et al. [17] reported that visual function is affected by astigmatism even when conventional VA is good, irrespective of the axes of astigmatism. Singh et al. [1] recently investigated the relationship between uncorrected astigmatism and VA in pseudophakia. They reported that uncorrected myopic astigmatism of more than 1 D results in a large loss of distance VA, without additional benefit to near VA, and that uncorrected hyperopic astigmatism results in deterioration of both distance and near VAs. They also showed that myopic astigmatism decreased VA by 0.31 (logMAR) per diopter regardless of the direction of the astigmatism axis and 0.23 (logMAR) per diopter for hyperopic astigmatism in emmetropic pseudophakia. Hasegawa et al. [18] reported that astigmatic defocus deteriorates contrast sensitivity.

The current study showed significant differences between monofocal and toric IOLs, with distant UCVA of 0.55 logMAR with monofocal IOLs and 0.38 logMAR with toric IOLs, 2 months postoperatively.

Therefore, implantation of a toric IOL is strongly recommended for patients with both cataract and corneal astigmatism [19, 20]. However, myopic patients who determined to nearsighted target such as -3D have to wear spectacle as usual after surgery, so the remaining astigmatism can be corrected by spectacle. Therefore, toric IOL is not actively recommended in such cases. However, these patients do not always wear spectacle at home and they often take them off when taking a shower or reading a book. The uncorrected visual acuity can be quite important considering that times without spectacle is not short at home.

According to a study by Nael et al. [21] on activity limitations due to decreased VA, 17.9 % or those whose distance Snellen VA is better than 20/63–20/60 (0.3–0.5 log MAR) have limitations with regard to activities of daily living (ADL) [22–26] such as dressing, washing, toileting, and eating, whereas nearly twice as many (32.2 %) of those who have VA worse than 20/63 (0.5 logMAR) have ADL limitations.

Myopia is a very common ophthalmologic disease, and its prevalence is increasing worldwide. Considering educational activities, continuous urbanization, and westernization, it is estimated that the prevalence of myopia will continue to increase [27, 28]. Myopic patients are increasingly undergoing cataract surgery; therefore, consultation on toric IOL with myopic target refraction is likely to increase. Based on the results of this study, we recommend toric IOL implantation in such patients to offer better quality of life after cataract surgery. This study is limited by its retrospective study design and short duration of follow up (2 month). And we did not measure subjective satisfaction after surgery in both groups. Therefore, further prospective studies with long observation period and new indicators such as quality of life questionnaires should be conducted to verify our results.

## Conclusions

In conclusion, implantation of a toric IOL was superior to monofocal IOL in terms of UCVA in both near and distance for cataract patients with astigmatism even when target refraction power is determined to myopia. To the best of our knowledge, this is the first study to quantify it. This may be helpful when an ophthalmologist consults patients. This study could be evidence in clinical practice to more actively recommend toric IOL when performing cataract surgery in patients with myopia.

## Data Availability

The data supporting our findings is contained in within the manuscript and tables. The datasets used and analyzed during the current study and literacy metrics are available from the corresponding author on reasonable request.

## Abbreviations

IOL: Intraocular lens
UCVA: Uncorrected visual acuity
BCVA: Best corrected visual acuity
LRI: Limbal relaxing incision
D: Diopter
LogMAR: Logarithm of the minimum angle of resolution
